# Evaluating Disparities in COVID‐19 Clinical Outcomes Among Patients With Cirrhosis in North America and Europe—An International Registry Study

**DOI:** 10.1002/jgh3.70064

**Published:** 2024-11-29

**Authors:** Umar Hayat, Andrew M. Moon, Manesh K. Gangwani, Fariha Hasan, Thomas Marjot, A. Sidney Barritt, Wasique Mirza, Duane Deivert, Muhammad Aziz, Dushyant Singh Dahiya, Hassam Ali, Sumant Inamdar, Mauricio Garcia‐Saenz‐de‐Sicilia

**Affiliations:** ^1^ Department of Internal Medicine Geisinger Wyoming Valley Medical Center Wilkes‐Barre Pennsylvania USA; ^2^ Division of Gastroenterology and Hepatology University of North Carolina at Chapel Hill Chapel Hill North Carolina USA; ^3^ Department of Gastroenterology and Hepatology University of Arkansas for Medical Sciences Little Rock Arkansas USA; ^4^ Department of Internal Medicine Cooper University Hospital Camden New Jersey USA; ^5^ Oxford Centre for Diabetes, Endocrinology and Metabolism, Radcliffe Department of Medicine University of Oxford Oxford UK; ^6^ Department of Gastroenterology and Hepatology Mercy Hospital Toledo Ohio USA; ^7^ Department of Gastroenterology and Hepatology University of Kansas Medical Center Kansas Missouri USA; ^8^ Department of Gastroenterology and Hepatology East Carolina University Greenville North Carolina USA; ^9^ Division of Gastroenterology and Hepatology University of Arkansas Little Rock Arkansas USA

**Keywords:** COVID‐19, COVID‐Hep, decompensated cirrhosis, racial disparities, SECURE‐Liver

## Abstract

**Background:**

Patients with decompensated cirrhosis have a higher risk of hospitalization, ICU admission, and death from COVID‐19. The impact of demographics on these outcomes remains uncertain.

**Methods:**

The SECURE‐Liver and COVID‐Hep databases were utilized to evaluate disparities in COVID‐19 outcomes. Patients were stratified by North American and European cohorts. Bivariate and multivariable logistic regression was performed.

**Results:**

A total of 718 cirrhosis patients with COVID‐19 were evaluated. In the North American cohort, Black patients had more comorbidities (CI: 1.86 vs. 1.83, *p* < 0.01), higher rates of hospitalization (77% vs. 85%, p < 0.01), ICU admission (27% vs. 40%, *p* = 0.05), and death (18% vs. 28%, *p* = 0.07). Hispanic patients had the lowest adverse outcome rates. In the European cohort, White patients had more comorbidities (CI; 1.63 vs. 1.31, *p* = 0.02), but non‐White patients had higher hospitalization rates (82% vs. 67%, *p* = 0.01), ICU admissions (15% vs. 18%, *p* = 0.04), and lower mortality rates (28% vs. 34%, p = 0.01).

**Conclusion:**

Black patients in North America had higher hospitalization, ICU admission, and death rates. In the European subgroup, White patients had higher death rates than non‐White patients. These disparities became statistically insignificant after adjusting for confounders, suggesting that non‐liver‐related comorbidities might increase the risk of adverse outcomes.

## 1 Introduction

The COVID‐19 pandemic has posed significant challenges to healthcare systems worldwide, with certain populations, including Black, Hispanic, and American Indians, experiencing a disproportionately high burden of severe illness and mortality [1, 2]. The adverse economic consequences and the onset of a recession during the COVID‐19 pandemic were exacerbated among populations at or below the poverty threshold and with already limited resources [3]. Among these vulnerable groups are patients with compensated and decompensated cirrhosis. Previous studies have established that patients with underlying liver diseases, including cirrhosis, are more susceptible to severe respiratory infections and poor outcomes from COVID‐19 [4, 5, 6, 7].

For several reasons, understanding the role of race and ethnicity in COVID‐19 outcomes among patients with decompensated cirrhosis is paramount. Firstly, there is increased awareness of substantial racial inequalities about COVID‐19, with Black and Hispanic populations having higher susceptibility to infection and mortality from the infection [8]. Studies using national death certificate data demonstrate a disproportionate impact of COVID‐19 in underrepresented racial/ethnic groups [5]. Moreover, the Centers for Disease Control (CDC) have reported a marked increase in the incidence of COVID‐19 cases, hospitalizations, and fatalities among Black, Hispanic, and American Indian communities when compared with White and non‐Hispanic individuals [9]. These existing health disparities associated with race and ethnicity may result from unequal access to healthcare services, quality of care, and social determinants of health [10]. Variations in housing, lifestyle, and nutrition [1], alongside genetic factors, immune response, and environmental exposures related to race and ethnicity, may also contribute to differential susceptibility to severe illness [11]. A better understanding of racial and ethnic disparities may inspire tailored approaches to care and other changes that could mitigate these disparities for ongoing and future pandemics. However, data regarding important confounders and patient outcomes is still scarce.

This study sought to determine the differences in COVID‐19 outcomes, including hospitalizations, intensive care unit (ICU) admission, and mortality, among patients with cirrhosis based on race/ethnicity.

## 2 Methodology

### 2.1 Data Source

We utilized the SECURE‐Liver and COVID‐Hep international databases to assess the influence of race and ethnicity on COVID‐19 outcomes among patients with cirrhosis. No clear information about cirrhosis etiology in individual patients was provided. These registries were developed from an online reported system of cases of laboratory‐confirmed SARS‐CoV‐2, as previously described [10, 11]. Clinicians were instructed to submit forms after patients had COVID‐19 long enough to experience recovery from infection or death.

SECURE‐Liver was supported by the American Association for the Study of Liver Diseases (AASLD) and COVID‐Hep by the European Association for the Study of the Liver (EASL). Data were stored at the University of North Carolina (SECURE‐Liver) and the University of Oxford (COVID‐Hep). Given that all data submitted to these registries were de‐identified, the University of Oxford Clinical Trials and Research Governance and the University of North Carolina Office of Human Research Ethics deemed this project was not human research, which did not require formal institutional review board approval.

### 2.2 Inclusion Criteria

Inclusion criteria were as follows: patient age ≥ 18 years old, presence of cirrhosis based on provider determination, positive PCR test for SARS‐CoV‐2, and case submission from North America or Europe.

### 2.3 Exposures and Covariates

The analyses were stratified based on continent: North America and Europe, given the inherited disparities in socioeconomic demographics and differences in racial/ethnic equalities. In North America, race/ethnicity was categorized as White/Non‐Hispanic, Black/Non‐Hispanic, and Hispanic, while in Europe, it was classified as White/Non‐Hispanic and Non‐White/Non‐Hispanic given the small number of patients of Black race. Clinicians who submitted online case report forms provided information on demographics, liver disease etiology and severity, and COVID‐19 outcomes. A comorbidities index (CI) was defined based on the presence of comorbidities associated with poor outcomes from COVID‐19, including diabetes mellitus, hypertension, obesity, smoking status, cardiovascular disease, and chronic kidney disease.

### 2.4 Statistical Analysis

Mean and standard deviations were calculated for quantitative variables. Qualitative variables were presented as absolute numbers and relative frequencies. Mean, standard deviation, and median were provided for normally distributed and interquartile range (IQR) skewed or ordinal scaled parameters. For qualitative variables, likelihood ratio chi‐square and Fisher's exact tests were conducted for 2 × 2 and rxc contingency tables to test the association between variables. Furthermore, the Cochran–Mantel–Haenszel test was used to identify associations between categorical/nominal variables while controlling for the strata variables in a multiway table. Bivariable logistic regression analysis assessed the odds of clinical outcomes by race/ethnicity, age, and sex. Subsequently, multivariable logistic regression was performed to assess clinical outcomes by race/ethnicity, adjusting for age, sex, comorbidity index, and Child Turcotte Pugh (CTP) classification status. In the multiple analyses, backward stepwise selection based on the probability of the Wald statistic was performed to detect parameters that might influence the outcome. A forward and backward analysis did not yield any statistical significance in outcomes.

A *p*‐value of < 0.05 was considered significant for all outcomes. Stata 17.0 software (Stata Corporation, College Station, TX, USA) was utilized for all data analyses.

## 3 Results

The registries included 718 patients with cirrhosis and COVID‐19 from North America and Europe. Among them, 290 were from North America and 428 from Europe.

### 3.1 North American Analysis

**FIGURE 1:**
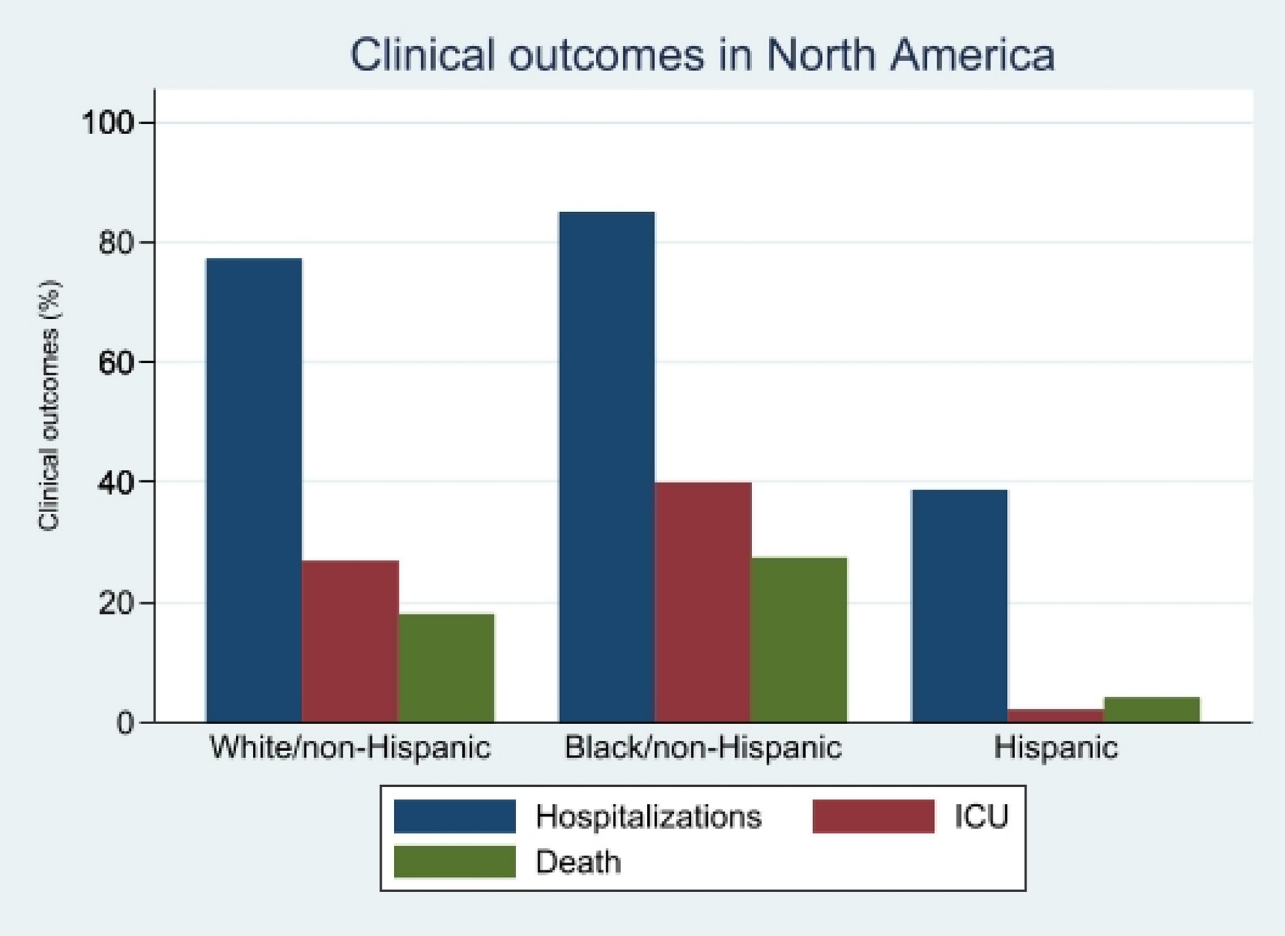
Hospitalization, ICU admissions, and deaths among White/non‐Hispanic, Black, and Hispanic populations in North America.

Baseline characteristics and outcomes can be found in Table 1. In the North American subgroup, 51% of patients were White/Non‐Hispanic, 14% were Black/Non‐Hispanic, and 17% were Hispanic. White/non‐Hispanic individuals had an average age of 61.3 years, while Black/Non‐Hispanic and Hispanic individuals were comparatively younger, with mean ages of 56.3 and 52.7 years, respectively. Regarding comorbidities, obesity was more prevalent in the White/Non‐Hispanic group (37%) than in the Black/Non‐Hispanic (30%) and Hispanic (12%) groups. Similarly, heart disease was more common in the White/Non‐Hispanic group (31%) compared with the Black/Non‐Hispanic group (24%). The prevalence of other comorbidities like hypertension and diabetes mellitus showed variations between groups. Child‐Pugh classification revealed differences in the severity of liver disease, with the Hispanic group having the highest proportion in the CTP A category (67.35%). Black patients had a higher prevalence of comorbidities than White patients with a Comorbidity Index (1.86 vs. 1.83, *p* = 0.007). Compared with White patients, a higher proportion of black patients were hospitalized (77% vs. 85%, *p* = 0.01), admitted to the ICU (27% vs. 40%, *p* = 0.05), and died (18% vs. 28%, *p* = 0.07) (Figure 1). However, in multivariable analyses, these differences were not statistically significant (Table 2).

**TABLE 1:** Baseline characteristics, comorbidities, and clinical outcomes of cirrhotic patients who tested positive for COVID‐19 in North America.

Characteristic	White/Non‐Hispanic (*N* = 149)	Black/ Non‐Hispanic (*N* = 40)	Hispanic (*N* = 49)
	*n* (%)	*n* (%)	*n* (%)
Age	61.30 ± 13.38	56.32 ± 13.67	52.65 ± 13.22
Sex—*N* (%)			
Male	103 (69.13)	18 (45)	24 (51)
Female	46 (30.87)	22 (55)	25 (49)
Comorbidities—*N* (%)			
Obesity	54 (37.24)	12 (30)	6 (12.24)
Heart disease	32 (31.37)	8 (23.53)	N/A
Hypertension	48 (47.06)	22 (64.71)	N/A
Diabetes mellitus	44 (43.14)	12 (35.29)	N/A
Chronic kidney disease	18 (17.65)	9 (26.47)	N/A
Smoking status	12 (11.71)	4 (11.76)	N/A
Child Pugh classification—*N* (%)			
CTP A	70 (46.98)	15 (37.50)	33 (67.35)
CTP B	44 (29.53)	13 (32.50)	16 (32.65)
CTP C	34 (22.82)	12 (30.00)	N/A
Ascites	15 (14.71)	11 (32.35)	N/A
Spontaneous bacterial peritonitis	1 (0.98)	2 (5.88)	N/A
Variceal bleeding	2 (1.96)	N/A	N/A
Outcomes			
Hospitalized	115 (77.18)	34 (85.00)	19 (38.78) p = 0.01
ICU admission	40 (26.85)	16 (40.00)	1 (2.04) p = 0.05
Ventilation	30 (20.13)	13 (32.50)	2 (4.08) p = 0.05
Death	27 (18.12)	11 (27.50)	2 (4.08) p = 0.07

**TABLE 2:** Multivariable models for clinical outcomes by race, age, and sex in North America.

Outcomes	White vs. Black odds ratio (95% CI)^a^	Hispanic odds ratio (95% CI)^a^	*p*
Hospitalization			
Race/ethnicity	1.41 (0.49–4.03)	0.70 (0.25–2.02)	0.52
Age, in 5‐year units	1.04 (0.89–1.23)		0.58
Sex: Male vs. female	0.73 (0.30–1.76)		0.48
ICU Admission			
Race/ethnicity	1.94 (0.80–4.67)	0.51 (0.21–1.24)	0.13
Age, in 5‐year units	1.01 (0.86–1.19)		0.81
Sex: Male vs. female	1.11 (0.50–2.46)		0.80
Death			
Race/ethnicity	1.25 (0.48–3.25)	0.79 (0.30–2.08)	0.64
Age, in 5‐year units	0.98 (0.39–2.17)		0.86
Sex: Male vs. female	0.92 (0.57–1.17)		0.26

^a^ Adjusting for comorbidity index (obesity, HTN, heart disease, diabetes, smoking status, chronic kidney disease, and Child Pugh classification status).

### 3.2 European Analysis

**FIGURE 2:**
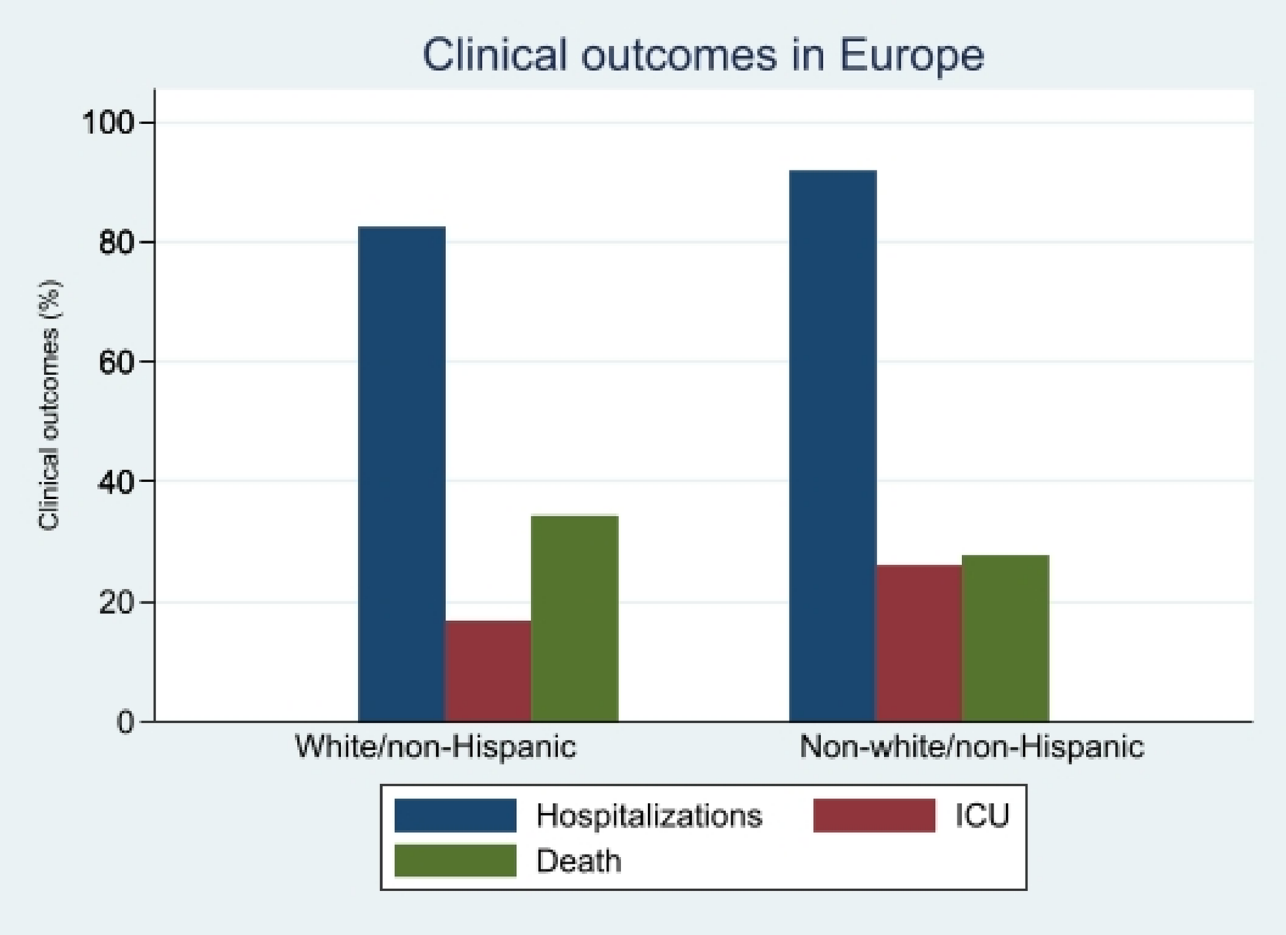
Hospitalization, ICU and deaths among White/non‐Hispanic, non‐White/non‐Hispanic population in Europe.

Baseline characteristics for this group can be found in Table 3. In the European subgroup analysis, 82% of patients were White/Non‐Hispanic, and 17% were Non‐White/Non‐Hispanic. The White/Non‐Hispanic group had an average age of 61.67 years, while the Non‐White/Non‐Hispanic group's average age was 57.60 years. Regarding health conditions, the White/Non‐Hispanic group had higher rates of obesity (32.25%), heart disease (23.34%), hypertension (39.37%), and diabetes mellitus (41.46%) compared with the Non‐White/Non‐Hispanic group, which had lower percentages for each of these conditions. Smoking and chronic kidney disease showed a similar pattern. In the Child‐Pugh classification, the Non‐White/Non‐Hispanic group had a slightly higher proportion in the CTP B category. Hospitalization rates were higher in the Non‐White/Non‐Hispanic group (91.78% vs. 82.37%), with relatively higher rates of ICU admission, ventilation, and death. Primary patient demographic characteristics, comorbidities, and clinical outcomes for both groups have been summarized in Table 3. White patients had a higher prevalence of comorbidities than Non‐White patients (CI; 1.63 vs. 1.31, *p* = 0.02). However, compared with White patients, a higher proportion of Non‐White patients were hospitalized (82% vs. 67%, *p* = 0.01) and admitted to the ICU (15% vs. 18%, *p* = 0.04), but fewer patients died (34.39% vs. 27.78%, p = 0.01) (Figure 2). In multivariable analysis, there was no statistically significant difference in the odds of hospital admission, ICU admission, or death between White/non‐Hispanic and Non‐White/non‐Hispanic groups (Table 4). See Figure 1 for outcomes in North America and Figure 2 for outcomes in Europe.

**TABLE 3:** Baseline characteristics, comorbidities, and clinical outcomes of the cirrhotic patients who tested positive for COVID‐19 in Europe.

Characteristic	White/Non‐Hispanic (*N* = 348)	Non‐White/ Non‐Hispanic (*n* = 73)
	*n* (%)	*n* (%)
Age	61.67 ± 12.80	57.60 ± 14.02
Sex—*N* (%)		
Male	243 (69.83)	54 (73.97)
Female	105 (30.17)	19 (26.03)
Comorbidities—*N* (%)		
Obesity	99 (32.25)	4 (9)
Heart disease	67 (23.34)	12 (16.67)
Hypertension	113 (39.37)	20 (27.78)
Diabetes mellitus	119 (41.46)	28 (38.89)
Chronic kidney disease	34 (11.85)	6 (8.33)
Smoking status	34 (11.85)	6 (8.33)
Child Pugh classification—*N* (%)		
CTP A	145 (41.98)	32 (44.44)
CTP B	115 (33.14)	26 (36.11)
CTP C	77 (22.1)	10 (13.89)
Ascites	81 (28.22)	16 (22.22)
Spontaneous bacterial peritonitis	8 (2.79)	N/A
Variceal bleeding	5 (1.74)	6 (8.33)
Outcomes		
Hospitalized	285 (82.37)	67 (91.78) *p* = 0.01
ICU admission	52 (15.03)	13 (17.81) *p* = 0.04
Ventilation	29 (8.41)	8 (10.96) *p* = 0.05
Death	119 (34.39)	20 (27.78) *p* = 0.01

**TABLE 4:** Multivariable models for clinical outcomes by race, age, and sex in Europe.

Outcomes	^a^Odds ratio (95% confidence interval)	*p*
Hospitalization		
Race: Non‐White vs. White	2.65 (0.76–9.29)	0.12
Age, in 5‐year units	1.05 (0.90–1.23)	0.49
Sex: Male vs. female	0.73 (0.32–1.66)	0.45
ICU admission		
Race: Non‐White vs. White	0.94 (0.38–2.32)	0.90
Age, in 5‐year units	0.82 (0.72–0.95)	0.08
Sex: Male vs. female	1.28 (0.63–2.61)	0.49
Death		
Race: Non‐White vs. White	0.72 (0.34–1.54)	0.40
Age, in 5‐year units	1.03 (0.92–1.15)	0.58
Sex: Male vs. female	0.70 (0.40–1.21)	0.20

^a^ Adjusting for comorbidity index (obesity, HTN, heart disease, diabetes, smoking status, chronic kidney disease, and Child Pugh classification status).

## 4 Discussion

This study evaluates the potential role of disparities by race and ethnicity among cirrhosis patients with COVID‐19 from North America and Europe. In both regions, racial differences in comorbidities and clinical outcomes were observed. Black patients in North America had more comorbidities and worse outcomes, but there was no statistical difference after adjusting for covariates. Similarly, non‐White patients had higher hospitalization and ICU admission rates in the European subgroups. However, these differences were not statistically significant in multivariable analysis.

There are established disparities based on race and ethnicity in health outcomes. Literature regarding disparities in COVID‐19 discusses the critical role of social determinants that have a dual impact: first, by directly influencing morbidity and mortality due to COVID‐19, and second, by their indirect influence through the development of comorbidities linked to adverse COVID‐19 outcomes [2]. Various factors at both the patient and hospital levels are essential to examine. Patient‐level factors include, but are not limited to, inherent distrust in the healthcare system and socioeconomic inequalities, which can lead to differences in insurance coverage and access to care, especially since the European population has universal healthcare access in one form or another [12]. Historical and structural inequities often predispose these disparities, which can have long‐term consequences for healthcare outcomes. Hospital‐based variables center around resource allocation and identified or unidentified biases within the healthcare system [13]. The distribution of resources can significantly impact the quality of care offered to patients of diverse racial and ethnic backgrounds, sustaining health inequities.

While our study is strengthened by representation from two continents, it is essential to exercise caution when making cross‐continental comparisons. There is significant country‐level as well as continent‐level variability of the influence of race and ethnicity on health outcomes. Ideally, our study would have been strengthened by including country of origin as a factor in our models. Unfortunately, this was not feasible due to the extensive number of countries involved and the relatively limited number of patients from many individual countries.

Nevertheless, the influence of race and ethnicity within continents did yield some notable findings. In our study, Black patients from North America had an increased percentage of comorbidities, including CTP C status, ascites, and subacute spontaneous bacterial peritonitis. All these conditions are related to worsened clinical outcomes. In our analysis, we performed forward and backward stepwise selection of covariates in multivariable models. This was done because many of these factors may serve as mediators, which should not be included in multivariable models, or confounders, which should be included. For instance, while medical comorbidities may be associated with both race (exposure) and COVID‐19 outcomes, it could be considered a confounder. However, these comorbidities are more heavily represented in individuals who are Black due to structural racism, which may lead to lower socioeconomic status and, consequently, greater levels of food inequality, unclean drinking water, air pollution, and stress [14, 15, 16]. Likewise, a similar argument can be applied to the assessment of cirrhosis severity, as systemic racism, healthcare provider biases, deferred specialist referrals, and other structural inequities contribute to delayed diagnosis and suboptimal management of cirrhosis among Black patients, including insufficient utilization of measures like spontaneous bacterial peritonitis (SBP) prevention [17, 18].

Structural racism persists, in part, due to the alignment of prejudiced practices across various systems. This results in interconnected systems inherently generating disparities in legislation and policymaking. These policies may ultimately foster discrimination, perpetuate stereotypes, and result in the unequal distribution of resources across key domains such as education, employment, housing, credit markets, healthcare, and the justice system [15].

Wang et al. [19] Highlighted healthcare disparities that are compounded by race/ethnicity. Various studies have also demonstrated disproportionately higher mortality rates from COVID‐19 in underserved communities, including Native Americans, Hispanics, and Blacks [2, 20], and our study similarly highlights this trend. Moreover, the lack of affordability, access, and awareness of COVID‐19 among minority non‐Hispanic blacks may have contributed to poor clinical outcomes among this population. In contrast, mortality was comparable between Whites, Blacks, and Hispanics in another study, with the highest mortality observed in Native Americans [21]. Previous studies have also noted disparities in COVID‐19 outcomes among Hispanic populations, with higher age‐adjusted hospitalization (1.5–8.6 times as high) and mortality (1.4–6.2 times as high) rates compared with their White counterparts [22, 23]. A lack of understanding of COVID‐19 vaccination's effectiveness among minority populations led to challenges during the vaccination campaign [24]. Special considerations such as culturally adapted vaccine awareness campaigns, early identification of crises among minorities, and simulation of appropriate responses for management could have addressed the routine healthcare needs of minority populations to mitigate these disparities in outcomes [24].

One of this study's strengths is its inclusion of a diverse population from Europe and North America. It also has access to granular clinical data unavailable in many studies examining this question.

This study had a few limitations. First, we needed more information on socioeconomic status, insurance coverage, and granular information on place of residence. Thus, we could not assess how these factors could further increase the risks of COVID‐19 infection and lead to worsened outcomes. There was also a low population percentage of Hispanics and Blacks in the European patient population, so further stratification between these two groups was not possible. It would have been beneficial to stratify cirrhosis further into differential causes, which could have provided valuable insights into lifestyle‐related factors contributing to the condition. Subsequently, this would have shed light on the complexities of cirrhosis and its relationship to race/ethnicity. Furthermore, no information is available in the database about the COVID‐19 vaccination status of the patients, which could have affected the study outcomes among different races/ethnicities based on their awareness and availability of the vaccines. Finally, the small number of outcomes may have also limited the possibility of detecting statistically significant differences in measured outcomes.

In conclusion, racial and ethnic disparities are essential components of social determinants of health. The variations observed in univariate and multivariable analyses may be linked to factors beyond liver‐related outcomes, including the influence of social determinants of health within the broader context of the pandemic's impact. These disparities are likely to have lasting effects on marginalized communities. Addressing these challenges requires a multifaceted approach encompassing healthcare access, quality, public health interventions, and policies to reduce health inequalities.

## Conflicts of Interest

A. Sidney Barritt IV and Andrew Moon are consultants for TARGET RWE.
